# The role of host eIF2α in viral infection

**DOI:** 10.1186/s12985-020-01362-6

**Published:** 2020-07-23

**Authors:** Yuanzhi Liu, Mingshu Wang, Anchun Cheng, Qiao Yang, Ying Wu, Renyong Jia, Mafeng Liu, Dekang Zhu, Shun Chen, Shaqiu Zhang, Xin-Xin Zhao, Juan Huang, Sai Mao, Xumin Ou, Qun Gao, Yin Wang, Zhiwen Xu, Zhengli Chen, Ling Zhu, Qihui Luo, Yunya Liu, Yanling Yu, Ling Zhang, Bin Tian, Leichang Pan, Mujeeb Ur Rehman, Xiaoyue Chen

**Affiliations:** 1 Institute of Preventive Veterinary Medicine, Sichuan Agricultural University, Wenjiang, Chengdu City, Sichuan 611130 P.R. China; 2 Key Laboratory of Animal Disease and Human Health of Sichuan Province, Sichuan Agricultural University, Wenjiang, Chengdu City, Sichuan 611130 P.R. China; 3 Avian Disease Research Center, College of Veterinary Medicine, Sichuan Agricultural University, Wenjiang, Chengdu City, Sichuan 611130 P.R. China

**Keywords:** Virus, eIF2α, General translation inhibition, Stress granule, Cell replication cycle, Autophagy/apoptosis

## Abstract

**Background:**

eIF2α is a regulatory node that controls protein synthesis initiation by its phosphorylation or dephosphorylation. General control nonderepressible-2 (GCN2), protein kinase R-like endoplasmic reticulum kinase (PERK), double-stranded RNA (dsRNA)-dependent protein kinase (PKR) and heme-regulated inhibitor (HRI) are four kinases that regulate eIF2α phosphorylation.

**Main body:**

In the viral infection process, dsRNA or viral proteins produced by viral proliferation activate different eIF2α kinases, resulting in eIF2α phosphorylation, which hinders ternary tRNA^Met^-GTP-eIF2 complex formation and inhibits host or viral protein synthesis. The stalled messenger ribonucleoprotein (mRNP) complex aggregates under viral infection stress to form stress granules (SGs), which encapsulate viral RNA and transcription- and translation-related proteins, thereby limiting virus proliferation. However, many viruses have evolved a corresponding escape mechanism to synthesize their own proteins in the event of host protein synthesis shutdown and SG formation caused by eIF2α phosphorylation, and viruses can block the cell replication cycle through the PERK-eIF2α pathway, providing a favorable environment for their own replication. Subsequently, viruses can induce host cell autophagy or apoptosis through the eIF2α-ATF4-CHOP pathway.

**Conclusions:**

This review summarizes the role of eIF2α in viral infection to provide a reference for studying the interactions between viruses and hosts.

## Background

Cellular DNA undergoes transcription, mRNA translation and processing/modification to form protein molecules with certain structures and functions. During this process, translation initiation is an important step in protein synthesis, which requires the participation of many eukaryotic initiation factors.

**Fig. 1:**
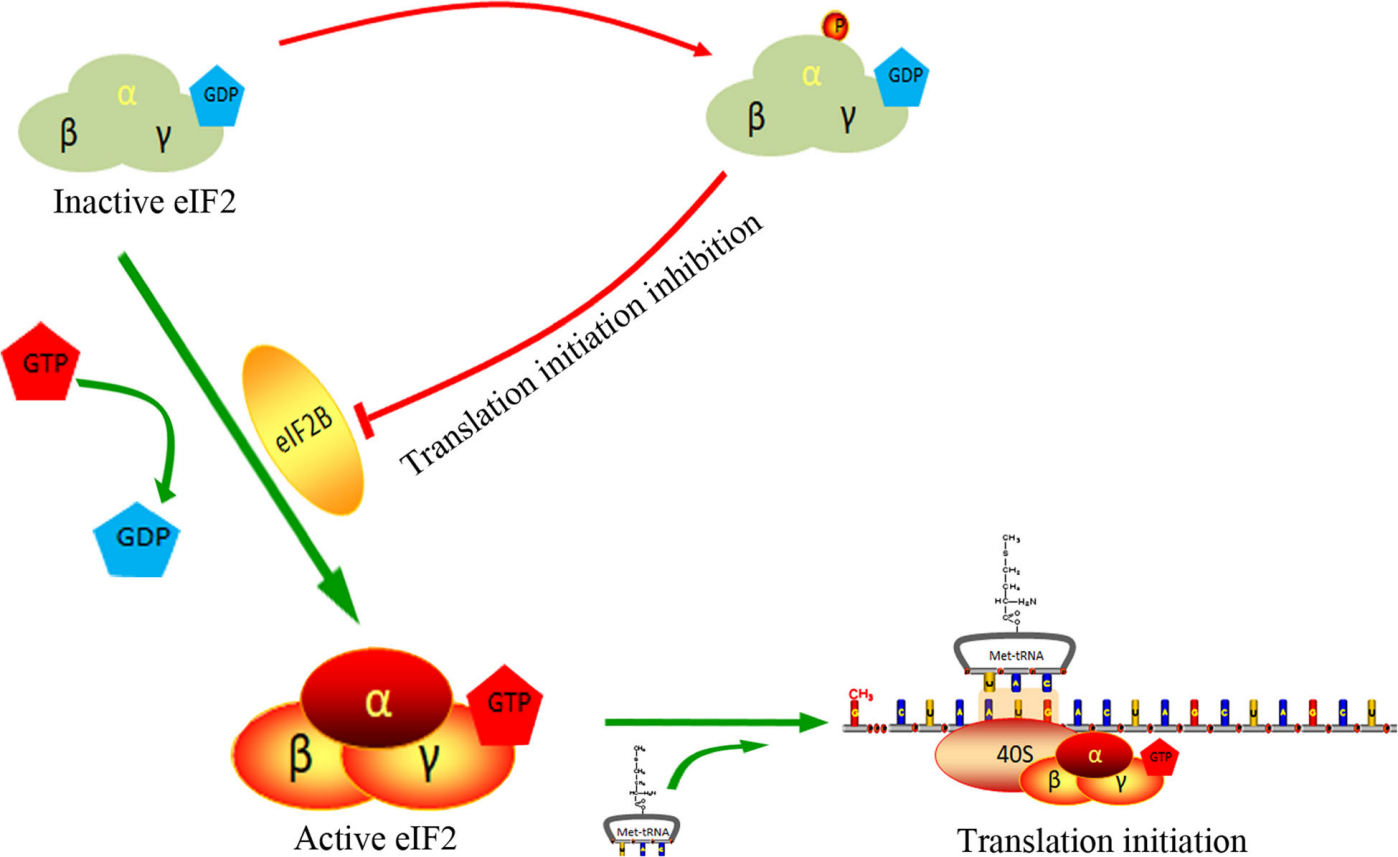
eIF2α phosphorylation inhibits translation initiation. The GTP conversion factor eIF2B converts inactive eIF2-GDP into active eIF2-GTP. The 43S complex containing eIF2-GTP scans along the mRNA for the initiation codon AUG. Once eIF2α is phosphorylated, the function of eIF2B to convert eIF2-GDP to eIF2-GTP is weakened or disappears, eventually leading to translational initiation inhibition [1–3]

eIF2, which is required for the initiation of most eukaryotic translation (Fig. 1), mediates the binding of Met-tRNAi^Met^ and the ribosomal 40S subunit in a GTP-dependent manner and initiates peptide chain synthesis. Under normal circumstances, the GTP conversion factor eIF2B converts inactive eIF2-GDP into active eIF2-GTP. Next, eIF2-GTP, Met-tRNAi^Met^, the ribosomal 40S subunit and other components (i.e., eIF1, eIF1A and eIF3) form the 43S complex, which scans for the initiation codon, AUG, along the mRNA. After the initiation codon is recognized, GTP bound to eIF2 is hydrolyzed to GDP by eIF5, and the initiation factors (eIF1, eIF2 and eIF5) bound to 40S dissociate (Fig. 4). Then, joining of the 60S large subunit mediated by eIF5B forms a complete initiation complex with the 40S small subunit and begins peptide chain synthesis. eIF2 is a heterotrimer composed of α, β and γ subunits, and its activity is regulated by phosphorylation of its α subunit at Ser51. Once eIF2α is phosphorylated, the eIF2 affinity for eIF2B is increased, and the ability of eIF2B to convert eIF2-GDP to eIF2-GTP is decreased or absent, resulting in GTP that cannot cycle, eventually inhibiting translational initiation [1–3]. In addition, the level of eIF2B in the cell is 10 to 20 times lower than that of eIF2; therefore, small changes in eIF2 phosphorylation can have a significant effect on protein translation [5].

Four kinases that phosphorylate eIF2α have been identified in mammals, namely, general control nonderepressible-2 (GCN2), protein kinase R-like endoplasmic reticulum kinase (PERK), double-stranded RNA (dsRNA)-dependent protein kinase (PKR), and heme-regulated inhibitor (HRI) [6]. These four kinases are all Ser/Thr kinases that phosphorylate Ser51 of the eIF2 α subunit under different stress conditions, which weakens the eIF2 ability to bind GTP during translation, resulting in the general translation suppression. After viral invasion, eIF2α phosphorylation is both advantageous and disadvantageous to cells: on the one hand, eIF2α phosphorylation shuts down protein synthesis and prevents viral replication; on the other hand, prolonged eIF2α phosphorylation leads to apoptosis or autophagy. In this paper, we discuss the role of eIF2α in viral infection and provide a reference for studying the interactions between viruses and hosts and the development of possible new targeted drugs.

## Main text

### Viral infection and eIF2α phosphorylation

The four host kinases mentioned above play different roles: PKR senses dsRNA during viral infection [7]; endoplasmic reticulum (ER) stress initiates the unfolded protein response (UPR) to activate PERK [8]; HRI monitors changes in hemoglobin levels [9, 10]; and GCN2 detects amino acid starvation [11], UV damage, and viral infection [12]. The main downstream substrate of these four kinases is eIF2α, but other substrates of these kinases have also been discovered. For instance, PERK acts on nuclear factor erythroid 2-related factor 2 (Nrf2) [13, 14], while PKR acts on nuclear factor kappa-light-chain-enhancer of activated B cells (NF-kB) [15, 16] and the tumor suppressor gene P53 [17]. This wide range of kinase activity contributes to the ability of different eIF2α kinases to sense different signals determined by unique regulatory characteristics. However, in some cases, the overlap of these stimuli leads to some redundancy between kinases [18]. After a virus invades the host cell, the host produces a corresponding stress response that stimulates stress kinases (GCN2, PKR, PERK, HRI), which affect eIF2α phosphorylation. Among these kinases, GCN2 and PKR have antiviral effects in the viral infection process. PERK plays a role in ER stress caused by viral protein synthesis, and HRI may play a role in the viral infection process. The four kinases play important roles in coordination under stress. However, under continuous viral infection, the virus promotes its own replication by regulating different eIF2α kinases.

#### Viral infection and GCN2

GCN2 activation, which occurs when cells lack essential amino acids, inhibits cellular protein translation [19, 20]. Recent studies have shown that GCN2 also plays an important role in inhibiting viral infection. GCN2 is involved in the innate antiviral response pathway of the host defense against RNA viruses, and mice in which GCN2 has been knocked out are more susceptible to Sindbis virus (SINV) and have higher viral titers in their brain than normal mice [21]. Human immunodeficiency virus-1 (HIV-1) activates GCN2, and GCN2 inhibits HIV-1 viral replication [22]. Similarly, GCN2 also has a strong inhibitory effect on vesicular stomatitis virus (VSV) [23]. Although GCN2 can inhibit viruses, viruses can also regulate GCN2. For example, HIV-1 encodes a protease (HIV-1Pro) that cleaves the viral precursor polyprotein, which cleaves GCN2 and prevents its antiviral effect [22]. The K3L protein of vaccinia virus (VV) interacts directly with the kinase catalytic domain of GCN2, inhibiting GCN2 and the general amino acid control pathway [24].

#### Viral infection and PERK

**Fig. 2:**
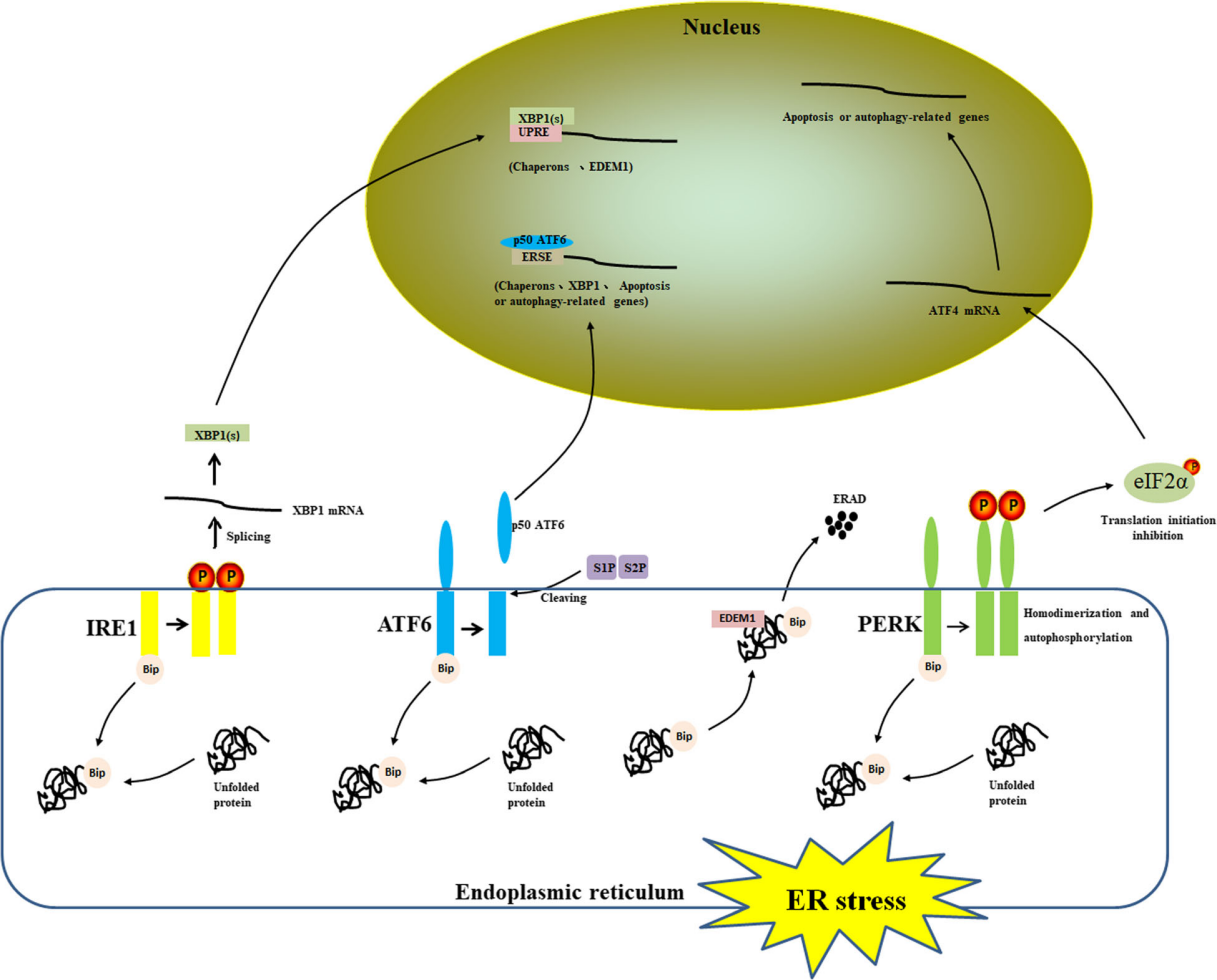
Three UPR signaling pathways. Under ER stress, ATF6 is processed to form active p50 ATF6, which is then transported to the nucleus to activate the transcription of genes containing an ERSE [25–27]. IRE1 cleaves 26 nucleotides from XBP1 mRNA to generate spliced XBP1 mRNA, which is translated into the active XBP1(s) protein. Subsequently, XBP1(s) activates the transcription of genes containing an UPRE [28–31]. Similarly, PERK is activated under cellular stress, which phosphorylates eIF2α and reduces protein synthesis to relieve ER stress [32, 33]

During viral infection, viral proteins pressure the host ER, resulting in ER stress. Host cells respond to ER stress, initiate the UPR, and activate the stress sensor PERK, inositol-requiring enzyme 1 (IRE1) and activating transcription factor 6 (ATF6). When the cell is at rest, these three stress sensors bind to glucose-regulated protein 78 (GRP78), which is also called binding immunoglobulin protein (Bip). Bip dissociates from the stress sensors and activates them in the presence of accumulated unfolded proteins in the ER (Fig. 2) [34, 35]. Under ER stress, ATF6 is released from Bip and transported to the Golgi complex, where it is sequentially cleaved by two proteases, the site 1 and site 2 proteases (S1P and S2P), to form active p50 ATF6; active p50 ATF6 is then transported to the nucleus, where it activates the transcription of genes containing the ER stress response element (ERSE), such as Bip and XBP1 [25–27]. Activated IRE1 cleaves 26 nucleotides from XBP1 mRNA to generate spliced XBP1 mRNA, which is translated to the active spliced XBP1 (XBP1(s)) protein. Subsequently, XBP1(s) activates the transcription of genes containing UPR elements (UPREs), such as ER-degradation-enhancing alpha-mannosidase-like protein-1 (EDEM1), which causes misfolded protein degradation [28–31]. Here, we focus on the importance of PERK in regulating ER stress and cell survival. This kinase phosphorylates the eIF2 α subunit and reduces protein synthesis to relieve ER stress [32, 33].

Most viruses stimulate the PERK-eIF2α pathway after infecting host cells, and there have been many reports on this process. Porcine reproductive and respiratory syndrome virus (PRRSV) phosphorylates eIF2α via a PERK-dependent mechanism and induces the formation of stress granules (SGs), which were shown to be involved in the PRRSV-induced inflammatory signaling pathway in MARC-145 cells [36]. In the late stage of PRRSV infection, PERK-mediated eIF2α phosphorylation inhibits tumor necrosis factor alpha (TNF-α) and interferon alpha (IFN-α) production in alveolar macrophages [37]. Hepatitis C virus (HCV) is a classic ER stress-inducing virus that activates three stressors and regulates this stress response [38–40]. The core protein and envelope proteins (E1 and E2) of HCV mature in the ER and are important proteins that cause ER stress [39–42]. However, HCV can modulate PERK kinase through the E1 and E2 proteins. E1 can interact with PERK, inhibit Bip and CHOP promoter activity and decrease CHOP expression induced by the UPR [40]. The E2 protein has a PKR-eIF2α phosphorylation site homology domain (PePHD), which can mimic PERK’s natural substrate eIF2α, thus reversing the translational repression induced by ER stress [39]. In addition, enterovirus 71 (EV71), porcine circovirus type 2 (PCV2), bluetongue virus type 1 (BTV1) and dengue virus (DENV) can also enhance replication using the PERK pathway [43–46]. PERK pathway activation increases Ca2+ and intracellular reactive oxygen species (ROS) levels in the cytosol and mitochondria to induce apoptosis [47].

#### Viral infection and PKR

The transcription of PKR, which has antiviral activity, is induced by IFN, and PKR is activated early in viral infection. In turn, IFN induction requires PKR catalytic activity, and PKR plays a role in the MDA5 signaling pathway [48]. PKR consists of an N-terminal dsRNA-binding domain (dsRBD) and a C-terminal kinase catalytic domain [49]. dsRNA produced during viral replication can bind the dsRBD at the N-terminus of PKR. dsRNA binding causes conformational changes that lead to dsRBD release from the kinase domain and induce PKR dimerization through the kinase domain and subsequent PKR phosphorylation [50]. Activated PKR inhibits viral protein translation primarily by phosphorylating eIF2α to achieve its antiviral effect.

Some viruses can use PKR activation to promote their replication. After the treatment of CHSE-214 and TO cells with the PKR inhibitor C16, infectious pancreatic necrosis virus (IPNV) induced reduced eIF2α phosphorylation, and the viral titer was decreased, indicating that IPNV proliferation depends on PKR activation [51]. Similarly, in HCV-infected cells, HCV replicated more efficiently only when PKR was activated, and PKR activation inhibition reduced the amount of virus [52]. One reason for this finding is that the NS5B protein interacts with PKR, and its RNA polymerase activity activates PKR, resulting in the decreased major histocompatibility complex I (MHC-I) expression [53]. Another reason for these effects is that HCV nonstructural protein 5A (NS5A) can directly bind the PKR protein kinase catalytic domain, thereby inducing IFN resistance [54].

**Fig. 3:**
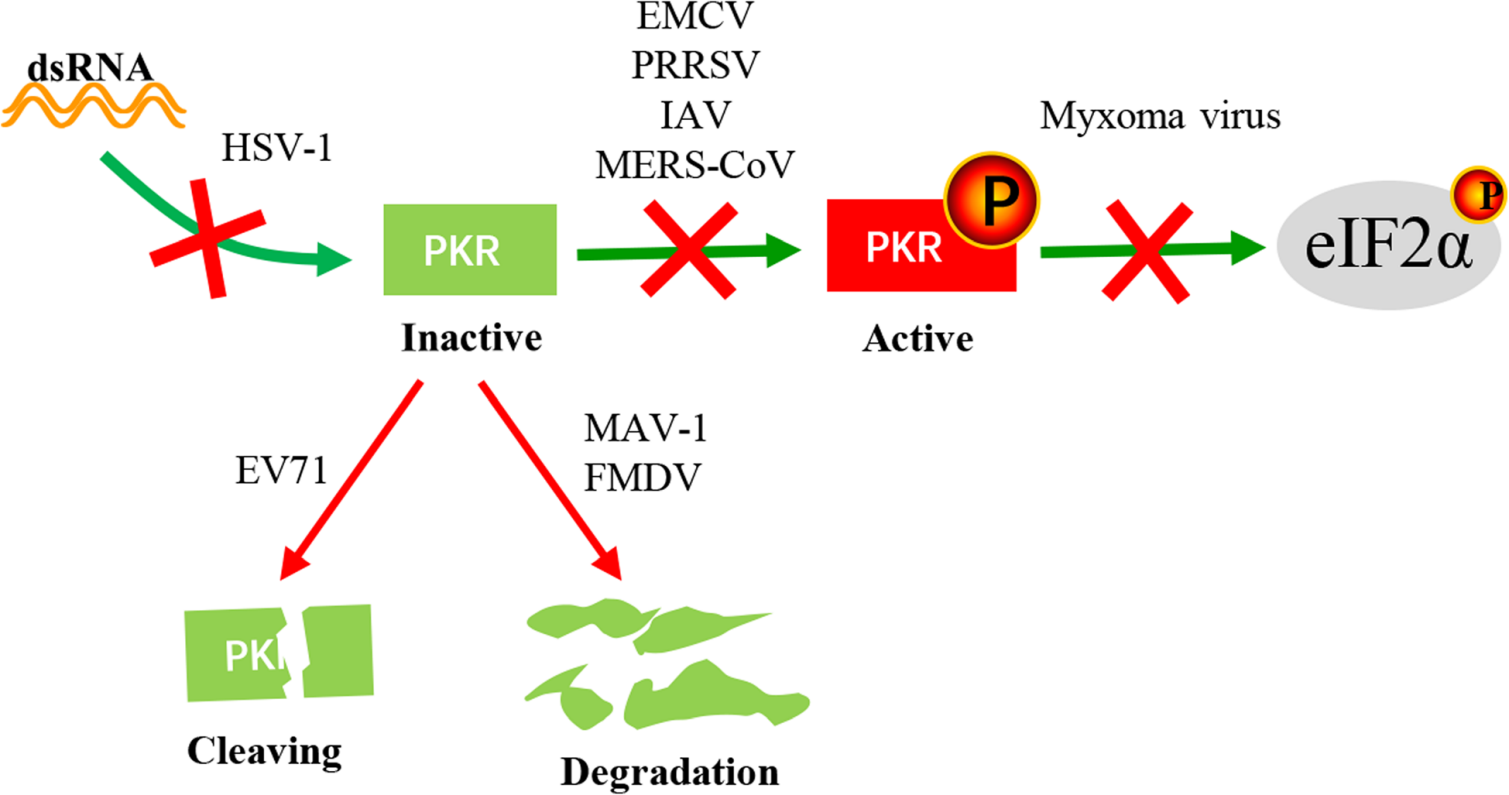
Viruses inhibit the antiviral effects of PKR. Viral dsRNA activates PKR, and activated PKR inhibits viral protein synthesis by phosphorylating eIF2α (green arrow). However, viruses have evolved many escape mechanisms (red arrows), such as hiding dsRNA (HSV-1 [55, 56]), blocking PKR activation (PRRSV [57], EMCV [58], IAV [59, 60], and MERS-CoV [61]), competing for PKR phosphorylation substrates (myxoma virus [62]), degrading PKR by the lysosome or proteasome pathway (MAV-1 [63] and FMDV [64]) and cleaving PKR via viral proteases (EV71 [65])

However, most viruses usually block the PKR antiviral effects in a variety of ways (Fig. 3). 1. Hiding dsRNA is one such strategy. A proline-rich structure on the RNA-binding protein (RBP) encoded by the Us11 gene of herpes simplex virus type 1 (HSV-1) inhibits PKR activation, and the RBP can bind viral dsRNA to avoid PKR recognition [55, 56]. 2. Blocking PKR activation is another strategy. Encephalomyocarditis virus (EMCV), PRRSV, the NS1 protein of influenza A virus (IAV) and the accessory protein 4a of Middle East respiratory syndrome coronavirus (MERS-CoV) prevent PKR activation and the effects of eIF2α phosphorylation on viral protein synthesis [57–61]. 3. Some viral factors act as a pseudosubstrate for PKR. The myxoma virus immunoregulatory protein M156R, an effective PKR phosphorylation substrate, competes with eIF2α, alleviating the effect of PKR on viral protein synthesis [62]. 4. Other viruses degrade PKR by the lysosome or proteasome pathway. Mouse adenovirus type 1 (MAV-1) and foot-and-mouth disease virus (FMDV) degrade PKR via the proteasome pathway and lysosome pathway, respectively [63, 64]. 5. Another strategy is PKR cleavage by viral proteases. After EV71 infection, the 3C protein can cleave PKR at position Q188, resulting in decreased PKR expression [65].

#### Viral infection and HRI

HRI was originally thought to be mainly expressed in nucleated red blood cells, where it regulates protein synthesis and mature erythrocyte numbers. However, recent studies have shown that HRI is also present in the liver and phagocytic cells [66]. HRI is inactive at normal hemoglobin concentrations; once hemoglobin concentrations are reduced, HRI can be activated by autophosphorylation [10]. The HRI heme-binding site is located at its N-terminus and kinase insert domain [67], containing H119, H120 and C409, which are required for HRI function [10]. HRI is known to play a protective role when the host is infected with bacteria [68, 69], but there have been few reports on the role of HRI during infections in mammals. HRI is the only activated stress kinase in arsenite-treated L929 cells, and its activation increases the protein expression and infection rate of reovirus in cells after arsenite treatment [70, 71].

Recently, HRI was reported to be involved in the fish immune response to viral infections. Rong et al. detected HRI mRNA transcription and protein expression in *Paralichthys olivaceus* immune organs and tissues, and HRI transcription and translation in the *P. olivaceus* head kidney and spleen tissues infected with turbot *Scophthalmus maximus* L. rhabdovirus (SMRV) were significantly enhanced [72]. Overexpression of HRI homologs from the orange-spotted grouper inhibited red-spotted grouper nervous necrosis virus (RGNNV) replication and increased IFN-associated cytokine levels [73].

### Effect of eIF2α phosphorylation on viral replication

#### eIF2α phosphorylation regulates host cell and virus translation

**Fig. 4:**
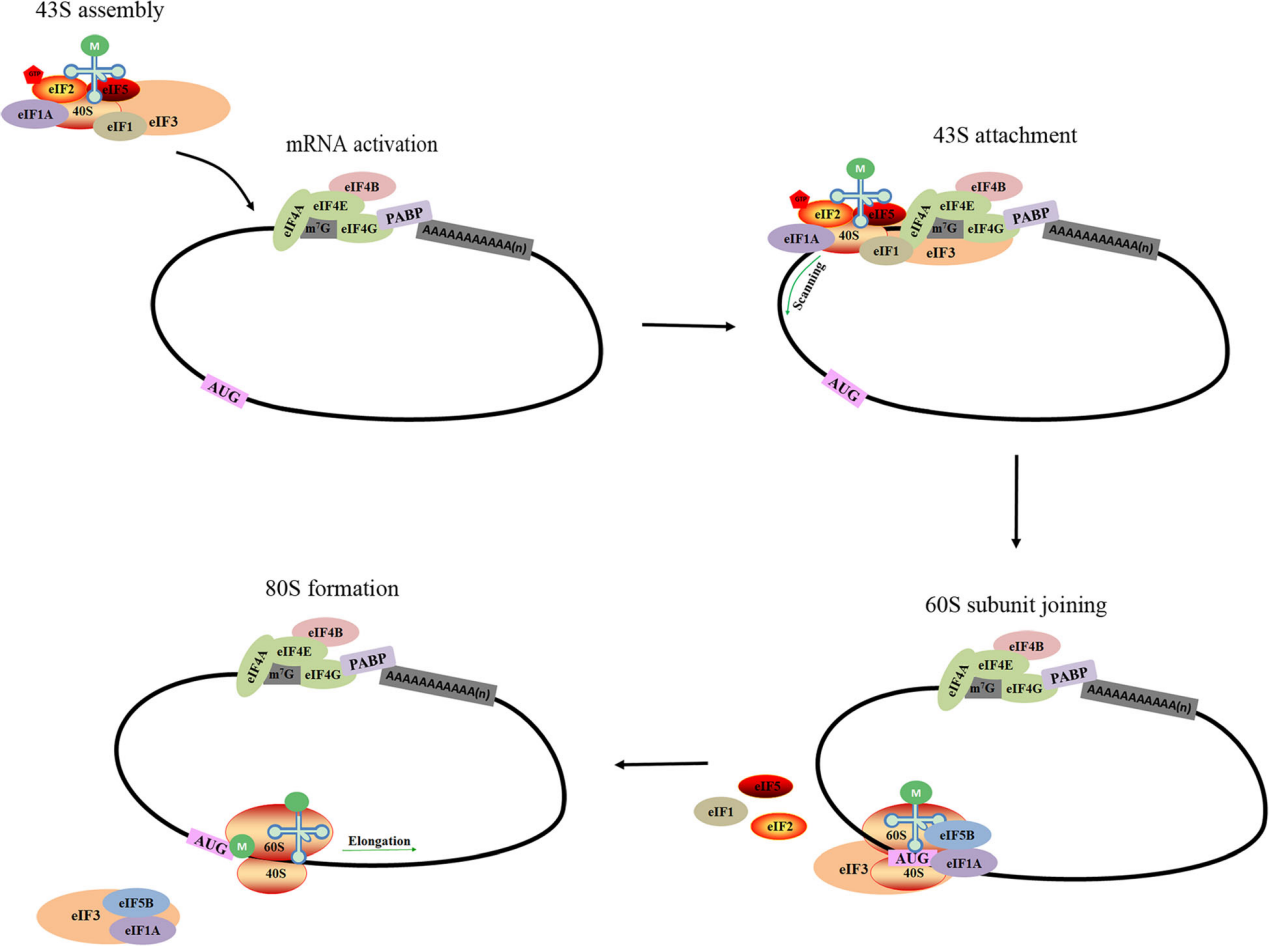
Classical cap-dependent translation initiation in eukaryotic cells. The process consists of 43S preinitiation complex assembly, 43S preinitiation complex binding to mRNA, initiation codon (AUG) recognition, and 60S ribosomal subunit addition to form a complete initiation complex and start translation [4]

Classic translation initiation in eukaryotic cells is dependent on the ribosome scanning mechanism of the cap structure (Fig. 4). The process occurs as follows: 43S preinitiation complex assembly, 43S preinitiation complex binding to mRNA, initiation codon (AUG) recognition, and 60S ribosomal subunit addition to form a complete initiation complex and initiate translation [4]. After the virus invades cells, it can hijack or disturb PABP, eIF4G, eIF4E, eIF2, etc., which are involved in classic cap-dependent translation initiation, and reduce the efficiency of intracellular mRNA recruitment by ribosomes [74–77]. Among these factors, eIF2 α subunit phosphorylation disrupts 43S subunit formation, leading to translation initiation cessation [78]. PRRSV infection induces host translational shutdown, which is associated with the C-terminal transmembrane (TM) region of viral nonstructural protein 2 (nsp2). PRRSV-induced host translational shutdown can be partially reversed by eIF2α dephosphorylation or mTOR pathway reactivation, suggesting that both eIF2α phosphorylation and mTOR signaling pathway attenuation contribute to PRRSV-induced host translational arrest [79]. However, not all viruses suppress ongoing host translation during their infection cycle [80]. For instance, the gamma [1] 34.5 protein of HSV-1 and the V16 and F18 residues in the African swine fever virus (ASFV) DP71L protein regulate protein phosphatase 1 (PP1) to dephosphorylate eIF2α, thereby avoiding general protein synthesis shutdown [81, 82]. Notably, DENV and MNV has been shown to trigger host cell translational shutdown uncoupled from eIF2α phosphorylation in recent findings [83–85], which may be a new strategy for inhibiting host translation, and the mechanism for inhibiting host translation requires further study.

**Fig. 5:**
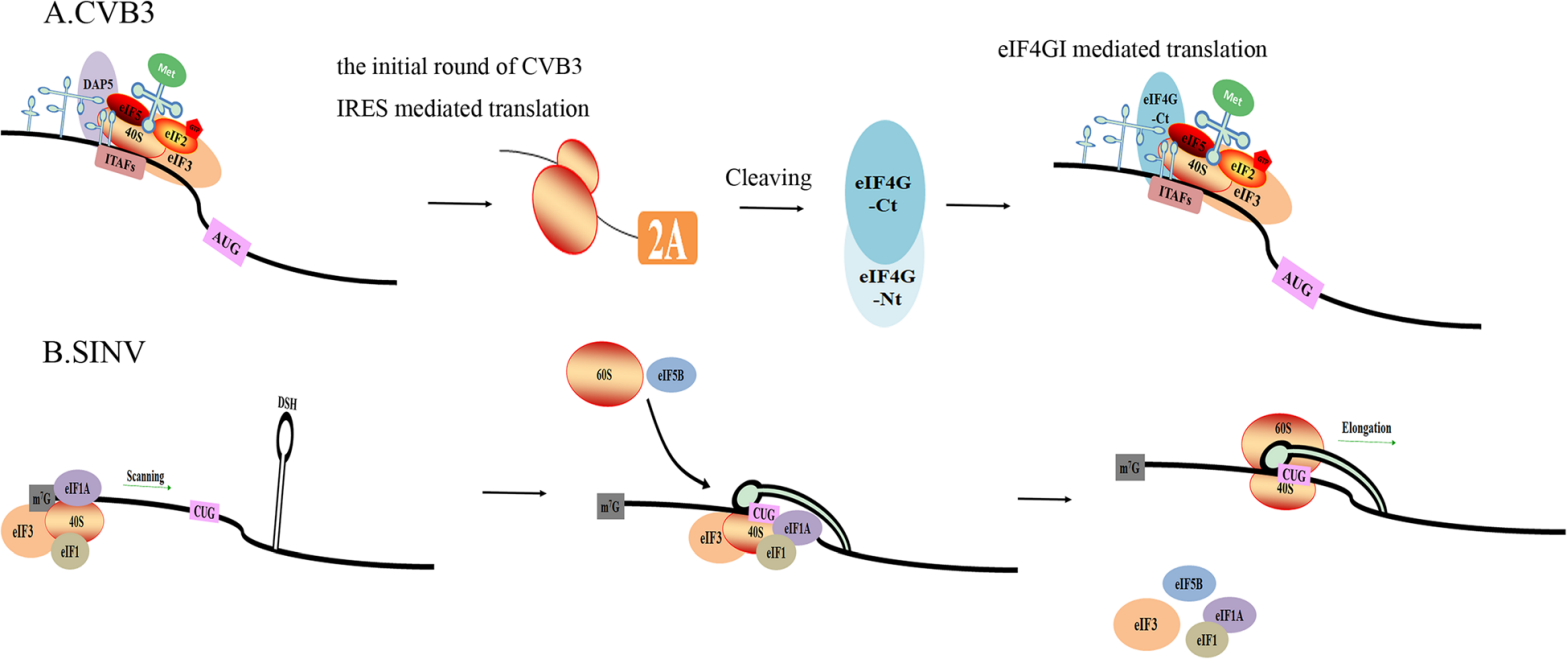
IRES-dependent and cap-independent translation initiation. **a** CVB3 RNA employs an IRES to recruit the 40S subunit to start translation. The host protein DAP5, some eIFs and ITAFs are required for IRES-driven translational initiation. Subsequently, the translated 2A protein cleaves eIF4GI into two parts. The N-terminus of eIF4GI recruited to the IRES effectively promotes viral replication [87]. **b** SINV contains a 5′ cap structure. The DSH structure on its sgRNA can initiate non-AUG codon translation to enhance viral protein synthesis [88, 89]

Differences in viral nucleic acids and their structures can lead to differences in their translation. The picornavirus genome does not have a 5′ cap structure but contains an internal ribosome entry site (IRES) sequence that can recruit a small ribosomal subunit to the viral mRNA translation initiation site with the help of some IRES-transacting factors (ITAFs) [86]. After coxsackievirus B type 3 (CVB3) enters the cell, it releases its viral nucleic acid and uses the host DAP5 protein to complete its first round of translation. Subsequently, the translated 2A protein cleaves eIF4GI into two parts. The eIF4GI N-terminus is recruited to the IRES sequence, effectively promoting viral replication (Fig. 5a) [87]. However, picornavirus infection can cause eIF2α phosphorylation, and translation may show a dual mechanism of eIF2 involvement: eIF2α phosphorylation was shown to block EMCV RNA translation in the early infection stages, synthesizing the proteins necessary for genome replication, whereas in the late EMCV infection phase, viral protein synthesis could occur in the presence of eIF2α phosphorylation [90]. In contrast, the SINV genome contains a 5′ cap structure. eIF2α phosphorylation weakens the translational function of the host, which should be inhibited as host mRNAs contain a 5′ cap structure; however, SINV mRNAs are all efficiently translated after eIF2α phosphorylation [91]. This phenomenon occurs because SINV subgenomic mRNA (sgRNA) has a downstream stable hairpin (DSH) structure located in the coding region, which facilitates non-AUG codon translation to enhance viral protein synthesis (Fig. 5b). The mutation of nucleotides on the DSH loop reduced translation initiation at the CUG codon, and DSH was shown to interact with the 40S subunit to facilitate sgRNA translation [88, 89].

How host translation initiation factors are used to promote the synthesis of a virus’s own proteins when eIF2α is phosphorylated is a controversial issue. It is suspected that viruses can use proteins other than eIF2 to promote translation of their proteins. Terenin et al. suggested that eIF2 was replaced by eIF5B and eIF3 in HCV-infected cells and that Met-tRNAi^Met^ was transported to the small ribosomal subunit independently of eIF2-GTP, thereby initiating viral IRES translation [92]. Kim et al. hypothesized that eIF2A can replace eIF2 and bind the HCV IRES to guide translation [93]. Subsequent studies have suggested that HCV mRNA initiates translation using the ternary Met-tRNAi^Met^-eIF2-GTP complex under normal cellular conditions but does not rely on eIF2 under stress because the interaction of the HCV IRES with the preinitiation complex replaces its interaction with eIF2 [94]. However, recent studies have shown that eIF2, eIF2A, eIF2D, eIF4A, and eIF4G are not involved in HCV IRES-driven translation [95]. Therefore, a deeper understanding of the viral translation mechanism is required. Changes in translation mechanisms after viral infection may be better understood through proteomics.

Although viruses can induce host translational shutdown, they can selectively express host mRNA, allowing the host to better serve the virus [80]. eIF2A or eIF2D can be used to replace eIF2 in the expression of these mRNAs or initiate non-AUG codon translation under stressful conditions due to viral infection [96]. During VV infection, the relative translational efficiency of mRNAs involved in oxidative phosphorylation, which is responsible for cellular energy production, was increased, and the levels and activities of proteins involved in oxidative phosphorylation were increased, while cellular oxidative phosphorylation inhibition significantly inhibited VV replication [97]. The alphavirus nsP3 protein YXXM motif binds the phosphatidylinositol 3-kinase (PI3K) regulatory subunit p85 and protein kinase B (PKB or AKT), increasing glucose metabolism toward fatty acid synthesis [98]. In addition, when eIF2α was phosphorylated, the stress-related proteins ATF4 and CHOP could still be synthesized [99]. The role of the eIF2α-ATF4-CHOP pathway in the virus life cycle is discussed in a later section.

#### eIF2α phosphorylation leads to SG formation

SGs are formed under stress (heat shock, starvation, ultraviolet radiation, or viral infection) [100]. SG assembly is driven by liquid-liquid phase separation (LLPS), which occurs when the collective interactions of a core protein-RNA interaction network breach a saturation threshold under stress. Ras GTPase-activating protein-binding protein 1 (G3BP1) is the central node of this network, which functions as a tunable switch to trigger RNA-dependent LLPS. Furthermore, G3BP1 can regulate the SG core network through positive or negative cooperativity with other G3BP1-binding factors [101].

The core component of SG formation is stalled preinitiation translation complex aggregation, including mRNA, the 40S ribosomal subunit, translation initiation factors and many RBPs [102]. G3BP1, T cell-restricted intracellular antigen 1 (TIA-1) and TIA-1-related protein (TIAR) are important RBPs in SGs. When cells adapt to stress or when the pressure is removed and the cells exhibit normal, balanced translation, SGs will disintegrate [103]. In some cases, SG formation is dependent on eIF2α phosphorylation [104], and phosphorylated eIF2α reduces the tRNAMet-GTP-eIF2 ternary complex activity level, which promotes TIA-1 transfer from the nucleus to the cytoplasm, where it binds the 48S complex to replace the ternary complex; these effects thus promote polyribosome decomposition and simultaneous mRNA transfer to SGs [105]. Since SGs isolate cellular and viral mRNAs and translational initiation factors to inhibit viral translation during viral infection, they are antiviral structures.

SG formation provides a platform for antiviral signaling pathways [58]. In the viral infection process, SGs can recruit a variety of proteins related to innate immunity, such as PKR, 2′-5′ oligoadenylate synthetase (OAS) and ribonuclease L (RNase L), to protect cells [106]. OAS-like protein 1 (OASL1), an OAS family member, is one of the SG components during viral infection. It contributes to type I IFN expression by trapping viral RNAs in SGs and increases the sensitivity of innate immune receptors involved in dsRNA recognition in the early infection stages [107]. Therefore, viral RNA and protein levels were found to be significantly inhibited in the early CVB3 infection stage [108].

Viruses have also evolved the following mechanisms to regulate SGs and thus promote their survival (Table 1). 1. Viruses change the cellular localizations of key proteins that make up SGs. The rotavirus VP2, NSP2 and NSP5 proteins can cause eIF2α phosphorylation but prevent SG formation by changing the localizations of the cellular proteins that make up SGs, allowing viral mRNA translation [5]. Mouse norovirus (MNV) and Zika virus (ZIKV) can recruit SG-related proteins into viral replication complexes to proliferate efficiently [84, 109]. 2. Viruses also cleave key proteins that make up SGs. This strategy is most common in picornaviruses. EV71 infection induces SG formation via the PKR-eIF2α pathway, but these SGs differ from typical SGs in their morphology and composition [118]. Later studies demonstrated that the EV71 2A protease cleaves eIF4GI to isolate cellular mRNA, allowing the virus to induce atypical SG formation and facilitate viral translation [110]. Another study also demonstrated that enterovirus (EV) 2Apro could antagonize SGs and inhibit IFN-β transcription [119]. However, Zhang et al. suggested that EV71 2Apro is a key viral component that triggers SG formation in the early infection stages. As infection progresses, SGs are destroyed due to G3BP1 cleavage mediated by the viral protease 3Cpro [111]. These seemingly contradictory results may be caused by different experimental methods. Similarly, the Theiler’s murine encephalomyelitis virus (TMEV) and FMDV L proteins can inhibit SG formation [113, 120]. Recent studies have shown that the picornavirus L and 2A proteins can interfere with the eIF4GI-G3BP1 interaction and block typical SG formation [114]. In addition, EMCV and poliovirus (PV) can inhibit SG formation by 3C-mediated cleavage of G3BP1 [58, 112]. These results show that the picornavirus L, 2A and 3C proteins are SG antagonist proteins. 3. Additionally, viruses hide their viral RNA. Human parainfluenza virus type 3 (HPIV3) induces SG formation by eIF2α phosphorylation and hides its newly synthesized viral RNA in inclusion bodies (IBs) to escape the antiviral effect of SGs [115]. 4. Moreover, viruses reduce eIF2α phosphorylation. The HIV-1 nucleocapsid (NC) interacts with the host dsRNA-binding protein Staufen1 to inhibit PKR and eIF2α phosphorylation, thereby dissociating SGs and relieving translation shutdown to achieve viral production [116, 117].

**Table 1:** Strategies of some viruses for inhibiting SG formation. Viruses have evolved the following strategies to regulate SGs and thus promote their survival

Virus	Viral protein	Strategies for resisting SG formation	Reference
Rotavirus		Changes the cellular localization of TIA-1 and PABP	[5]
MNV		Recruits G3BP1 to the viral replication complex	[84]
ZIKV		Alters the cellular localization of HuR	[109]
EV71	2A	Cleaves eIF4GI	[110]
EV71	3C	Cleaves G3BP1	[111]
PV	3C	Cleaves G3BP1	[112]
FMDV	L	Cleaves G3BP1 and G3BP2	[113]
EMCV	3C	Cleaves G3BP1	[58]
Picornavirus	L or 2A	Interferes with the eIF4GI-G3BP1 interaction	[114]
HPIV3		Hides viral RNA	[115]
HIV-1		Inhibits PKR and eIF2α phosphorylation	[116, 117]

#### eIF2α phosphorylation blocks the cell replication cycle

The cell replication cycle is divided into three phases: the G0 phase at rest, the intermitotic phase (G1 phase, S phase, and G2 phase) and the mitosis phase (M phase). After a virus invades cells, it blocks the cell cycle. Some viruses block the cell cycle at the G/M phase [121, 122], while some viruses block the cell cycle at the G0/G1 phase. G0/G1 phase blockade is associated with eIF2α phosphorylation [123]. Newcastle disease virus (NDV) infection activated the PERK-eIF2α-CHOP pathway in HeLa cells, downregulating cyclin D1 to arrest the cell cycle at the G0/G1 phase and providing a favorable environment for NDV replication [124]. The Muscovy duck reovirus (MDRV) P10.8 protein reduced cyclin-dependent kinase 2 (CDK2), cyclin-dependent kinase 4 (CDK4) and cyclin E expression in DF-1 cells via the PERK-eIF2α pathway, arresting the cells in the G0/G1 phase [125]. The virus replication process requires a series of proteins in the host cell that facilitate its replication, and viral infection causes the cells to stagnate in the G0/G1 phase, reducing the competitive pressure due to intracellular DNA replication and providing an environment favorable for virus replication [126, 127].

#### eIF2α phosphorylation mediates cell autophagy or apoptosis

**Fig. 6:**
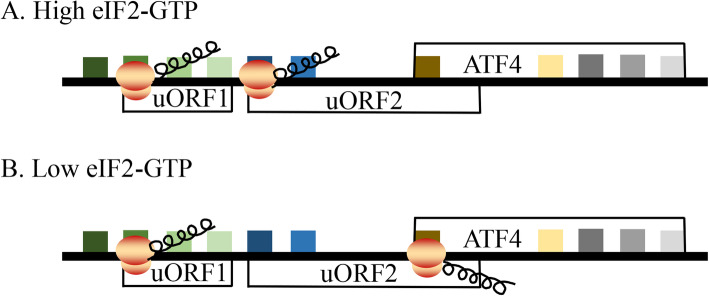
The translation regulation of ATF4 under stress conditions. ATF4 translation is regulated through upstream open reading frames (uORFs). The 5′-proximal uORF1 encodes three amino acid residues, and the uORF2 and ATF4 coding regions partially overlap. In nonstressed cells, uORF1 facilitates ribosome scanning and reinitiates at the downstream coding region, uORF2, which blocks ATF4 expression. Under stress conditions, the time required for scanning ribosomes to reinitiate translation increases; thus, uORF1 allows ribosomes to scan through uORF2 and to initiate the ATF4-coding region [129]

Under stress, the host can regulate autophagy via the eIF2α kinase signaling pathway [128]. Activating transcription factor 4 (ATF4) is preferentially translated during eIF2α phosphorylation. ATF4 has two upstream open reading frames (uORFs) that regulate ATF4 translation (Fig. 6). uORF1 is a positive element that facilitates ribosome scanning and reinitiation at downstream coding regions in ATF4 mRNA, while uORF2 is an inhibitory element that blocks ATF4 expression. In nonstressed cells, uORF1 facilitates ribosome scanning and reinitiates at the downstream coding region, uORF2, which blocks ATF4 expression. Under stress conditions, the time required for scanning ribosomes to reinitiate translation increases; thus, uORF1 allows ribosomes to scan through uORF2 and to initiate the ATF4-coding region [129]. ATF4 regulates ATF4-specific target genes through heterodimerization or posttranslational modifications (ubiquitination, phosphorylation, acetylation, or methylation), and these target genes are involved in apoptosis or autophagy, the cell cycle, amino acid import and metabolism, and resistance to oxidative stress [130–137]. Therefore, ATF4 is the main stress regulator of cells, balancing the pro- and anti-survival of cells [138]. Here, we discuss that viral infection induces autophagy or apoptosis through the eIF2α-ATF4 pathway.

Under stress, the eIF2α-ATF4 pathway activates the transcription of a large number of autophagy genes (P62, Atg16l1, Map1lc3b, Atg12, Atg3, Becn1, Gabarapl2, etc.) [139]. Autophagy enables cells to recover amino acids and nutrients to maintain protein synthesis, energy synthesis and metabolic balance [140]. After viral invasion, autophagy can process and present antigens to MHC-I and MHC-II molecules in order to activate adaptive immune responses [141–143]. However, the virus can regulate autophagy to facilitate its own replication [144–146]. The FMDV capsid protein VP2 was found to activate the eIF2α-ATF4 pathway and interact with heat shock protein beta-1 (HSPB1) to induce autophagy, while FMDV replication was significantly reduced when autophagy was suppressed [145]. Mammalian cell infection with HSV-1 induced autophagy via PKR-eIF2α, but this autophagy could be antagonized by the HSV-1 neurovirulence gene product ICP34.5 [128]. Virus-induced autophagy is often associated with ER stress. For example, DENV, CVB3, duck enteritis virus (DEV), prototype foamy virus (PFV) and other viruses induce autophagy through the PERK-eIF2α pathway [44, 147–149].

CHOP, a key ATF4 downstream target, is also efficiently translated when eIF2α is phosphorylated. It can be speculated that CHOP may have a translation regulation mechanism similar to that of ATF4. However, viral infection prolongs stress, and CHOP is involved in apoptosis. PCV2, the MDRV p10.8 protein and the Japanese encephalitis virus (JEV) NS4B protein promote apoptosis via the PERK-eIF2α-ATF4-CHOP pathway [46, 125, 150, 151]. Similarly, human astroviruses (HAstVs) activate caspase using the eIF2α-ATF4-CHOP pathway for viral release [152]. Interestingly, West Nile virus (WNV) proliferated to significantly higher viral titers in CHOP-deficient mouse embryonic fibroblasts (MEFs) than in wild-type MEFs, which indicates that CHOP-mediated apoptosis functions to control WNV replication [153]. This finding shows that apoptosis has a dual role in viruses: on the one hand, apoptosis is beneficial for viral replication and release; on the other hand, apoptosis of host cells can inhibit viral spread to protect uninfected cells. Regarding the role of the latter, the virus can regulate CHOP expression for survival. The ASFV DP71L protein recruits PP1c to dephosphorylate eIF2α and inhibit ATF4 and downstream CHOP, although the DP71L gene is not the only factor required for ASFV to control eIF2α phosphorylation during infection [154]. The hepatitis B virus (HBV) X protein interacts directly with GRP78, inhibiting eIF2α phosphorylation and subsequently inhibiting ATF4-CHOP-Bcl-2 expression to prevent hepatocellular carcinoma (HCC) cell death and the negative regulation of DNA repair [155].

CHOP can activate growth arrest and DNA damage-inducible protein (GADD34) and PP1 to promote eIF2α dephosphorylation and restore protein translation [156–158]. In the late NDV infection stage, eIF2α phosphorylation leads to host cell translational shutdown, and GADD34 levels are upregulated, but PP1 downregulation counteracts the role of GADD34 [159]. The HSV γ34.5 protein and the ASFV DP71L protein exhibit sequence similarity with GADD34, and PP1 recruitment dephosphorylates eIF2α in infected cells, thereby promoting viral protein synthesis [160].

## Conclusion

**Fig. 7:**
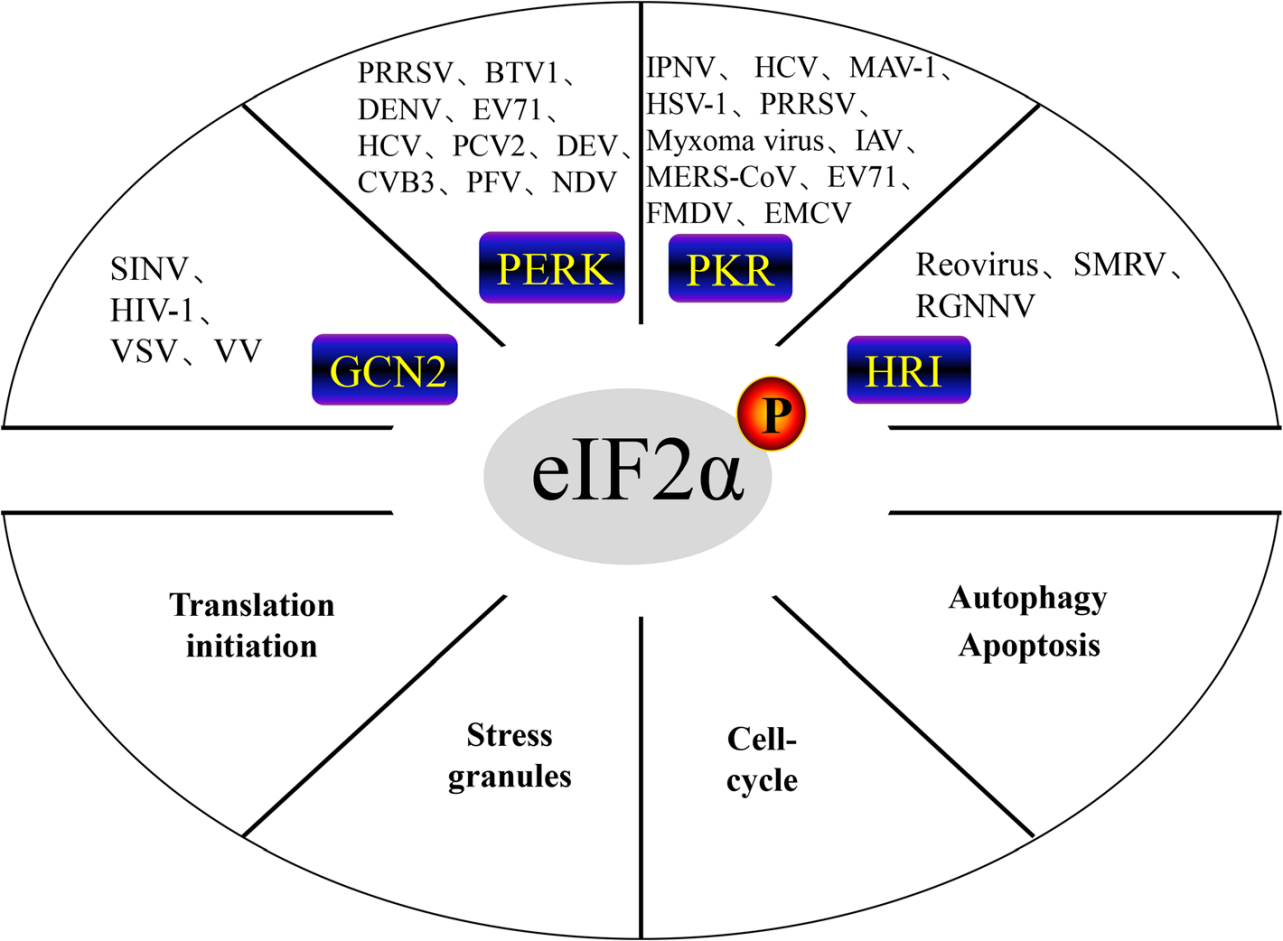
The role of host eIF2α in viral infection. Different viruses can stimulate specific eIF2α kinases, or the same virus can stimulate different eIF2α kinases, such as PRRSV, HCV, and EV71, which can activate PKR and PERK. eIF2α phosphorylation can affect host cell translation efficiency, SG formation, the cell cycle, and autophagy or apoptosis, thus facilitating viral infection

After viruses infect a host, they affect eIF2α phosphorylation to promote self-replication. This process is manifested in main four ways (Fig. 7). First, the kinase-promoted eIF2α phosphorylation inhibits host protein translation, and proteins beneficial to the virus itself are selectively expressed. Some viruses inhibit eIF2α phosphorylation via GADD34 or PP1, avoiding host protein synthesis shutdown and promoting viral protein synthesis. Second, eIF2α phosphorylation leads to SG formation, which encapsulate host and viral mRNAs and some translation initiation factors, but viruses can evade the antiviral effect of SGs in different ways, thereby promoting self-replication. Third, viruses use the PERK-eIF2α-CDK pathway to induce cell cycle arrest, providing a favorable environment for their replication. Fourth, viral infection leads to abnormal kinase expression, and autophagy or apoptosis is regulated by the eIF2α-ATF4 pathway to promote viral proliferation.

eIF2α, a balance point between cellular resistance to viruses and virus-induced apoptosis or autophagy, is essential for cell survival. Anne Bertolotti et al. found that the phosphatase regulatory subunit PPP1R15A (R15A) inhibitor Sephin1 could increase the eIF2α phosphorylation level [161], and studies have shown that Sephin1 has inhibitory effects on some RNA and DNA viruses [162]. In addition, pathologic changes caused by viruses can induce cancerous changes in cells, such as HCC development in patients with HCV infection [163, 164]. Some studies have shown that eIF2α phosphorylation inhibits c-Myc-mediated glycolysis, thereby inhibiting cancer growth and metastasis [165, 166]. Therefore, the study of eIF2α has played an important role in revealing viral pathogenesis and new targeted drug development. Simultaneously, how viruses synthesize their own proteins in the case of eIF2α phosphorylation also needs to be further clarified systematically.

## Data Availability

Not applicable.

## Abbreviations

GCN2: General control nonderepressible-2
PERK: Protein kinase R-like endoplasmic reticulum kinase
PKR: Protein kinase double-stranded RNA-dependent
HRI: Heme-regulated inhibitor
mRNP: Messenger ribonucleoprotein
SGs: Stress granules
eIF: Eukaryotic initiation factor
ER: Endoplasmic reticulum
UPR: Unfolded protein response
Nrf2: Nuclear factor erythroid 2-related factor 2
NF-kB: Nuclear factor kappa-light-chain-enhancer of activated B cells
SV: Sindbis virus
HIV-1: Human immunodeficiency virus-1
VSV: Vesicular stomatitis virus
VV: Vaccinia virus
IRE1: Inositol-requiring enzyme 1
ATF6: Activating transcription factors 6
GRP78: Glucose-regulated protein 78
Bip: Binding immunoglobulin protein
ERSE: ER stress response element
EDEM1: ER-degradation-enhancing alpha-mannosidase-like protein-1
PRRSV: Porcine reproductive and respiratory syndrome virus
TNF-α: Tumor necrosis factor alpha
IFN-α: Interferon alpha
BTV1: Bluetongue virus type 1
DENV: Dengue virus
EV71: Enterovirus 71
HCV: Hepatitis C virus
PePHD: PKR-eIF2α phosphorylation site homology domain
PCV2: Porcine circovirus type 2
ROS: Reactive oxygen species
dsRBD: dsRNA-binding domain
IPNV: Infectious pancreatic necrosis virus
MAV-1: Mouse adenovirus type 1
HSV-1: Herpes simplex virus type 1
IAV: Influenza A virus
MERS-CoV: Middle East respiratory coronavirus
FMDV: Foot-and-mouth disease virus
EMCV: Encephalomyocarditis virus
SMRV: *Scophthalmus maximus* L. rhabdovirus
RGNNV: Red-spotted grouper nervous necrosis virus
PP1: Protein phosphatase 1
ASFV: African swine fever virus
IRES: Internal ribosome entry site
ITAFs: IRES transacting factors
CVB3: Coxsackievirus B type 3
sgRNA: Subgenomic mRNA
DSH: Downstream stable hairpin
PI3K: Phosphatidylinositol 3-kinase
PKB or AKT: Protein kinase B
RBPs: RNA-binding proteins
G3BP1: Ras GTPase-activating protein-binding protein 1
TIA-1: T cell-restricted intracellular antigen 1
TIAR: TIA-1-related protein
OAS: 2′-5′ oligoadenylate synthetase
RNase L: Ribonuclease L
RLR: Retinoic acid-inducible gene I-like receptor
MNV: Mouse norovirus
ZIKV: Zika virus
TMEV: Theiler’s murine encephalomyelitis virus
PV: Poliovirus
HPIV3: Human parainfluenza virus type 3
IBs: Inclusion bodies
NDV: Newcastle disease virus
MDRV: Muscovy duck reovirus
CDK2: Cyclin-dependent kinase 2
CDK4: Cyclin-dependent kinase 4
ATF4: Activating transcription factor 4
MHC-I: Major histocompatibility complex I
MHC-II: Major histocompatibility complex II
HSPB1: Heat shock protein beta-1
DEV: Duck enteritis virus
PFV: Prototype foamy virus
Bcl-2: B-cell lymphoma 2
HAstVs: Human astroviruses
JEV: Japanese encephalitis virus
HBV: Hepatitis B virus
HCC: Hepatocellular carcinoma
GADD34: Growth arrest and DNA damage-inducible protein
